# From simple and specific zymographic detections to the annotation of a fungus *Daldinia caldariorum* D263 that encodes a wide range of highly bioactive cellulolytic enzymes

**DOI:** 10.1186/s13068-021-01959-1

**Published:** 2021-05-21

**Authors:** Meng-Chun Lin, Hsion-Wen Kuo, Mu-Rong Kao, Wen-Dar Lin, Chen-Wei Li, Kuo-Sheng Hung, Sheng-Chih Yang, Su-May Yu, Tuan-Hua David Ho

**Affiliations:** 1 Institute of Plant and Microbial Biology, Academia Sinica, Taipei, Taiwan, ROC; 2 Institute of Molecular Biology, Academia Sinica, Taipei, Taiwan, ROC; 3 Department of Environmental Science and Engineering, Tunghai University, Taichung, Taiwan, ROC; 4 Institute of Tropical Plant Science, National Cheng Kung University, Tainan, Taiwan, ROC; 5 Biotechnology Research Center, National Chung Hsing University, Taichung, Taiwan, ROC

**Keywords:** Zymography, Genomic annotation, Biomass degradation, Cellulolytic enzymes

## Abstract

**Background:**

Lignocellulolytic enzymes are essential for agricultural waste disposal and production of renewable bioenergy. Many commercialized cellulase mixtures have been developed, mostly from saprophytic or endophytic fungal species. The cost of complete cellulose digestion is considerable because a wide range of cellulolytic enzymes is needed. However, most fungi can only produce limited range of highly bioactive cellulolytic enzymes. We aimed to investigate a simple yet specific method for discovering unique enzymes so that fungal species producing a diverse group of cellulolytic enzymes can be identified.

**Results:**

The culture medium of an endophytic fungus, *Daldinia caldariorum* D263, contained a complete set of cellulolytic enzymes capable of effectively digesting cellulose residues into glucose. By taking advantage of the unique product inhibition property of β-glucosidases, we have established an improved zymography method that can easily distinguish β-glucosidase and exoglucanase activity. Our zymography method revealed that D263 can secrete a wide range of highly bioactive cellulases. Analyzing the assembled genome of D263, we found over 100 potential genes for cellulolytic enzymes that are distinct from those of the commercially used fungal species *Trichoderma reesei* and *Aspergillus niger*. We further identified several of these cellulolytic enzymes by mass spectrometry.

**Conclusions:**

The genome of *Daldinia caldariorum* D263 has been sequenced and annotated taking advantage of a simple yet specific zymography method followed by mass spectrometry analysis, and it appears to encode and secrete a wide range of bioactive cellulolytic enzymes. The genome and cellulolytic enzyme secretion of this unique endophytic fungus should be of value for identifying active cellulolytic enzymes that can facilitate conversion of agricultural wastes to fermentable sugars for the industrial production of biofuels.

**Supplementary Information:**

The online version contains supplementary material available at 10.1186/s13068-021-01959-1.

## Background

Because of the rapid development of our society in recent decades, our demands on liquid fuels have been increasing, and current reserves of fossil fuels will be exhausted in the near future. Thus, improvements in alternative energy have become imperative to sustain the further development of our society. First-generation starch-based biofuels may supplement our energy requirement, but are not sustainable because of the use of food commodities as the starting raw material. Second-generation biofuels are produced from decomposition of lignocellulosic biomass without competition with our food resources and are thus more sustainable than first-generation biofuels. The major sources of second-generation biofuels are agricultural wastes such as rice straw and corn stover that contain high amounts of lignocellulose [1]. However, lignocellulose needs to be converted into sugars that can be further fermented into biofuels. Current lignocellulose can be decomposed by thermochemical and biochemical processes. The thermochemical routes, such as pyrolysis and gasification [2, 3], decompose agricultural wastes into a mixture of gaseous and liquid forms of hydrocarbons. Although multiple compounds can be acquired by the thermochemical route, the processing requires high temperature and thus depends on high energy input. However, the biochemical route utilizes lignocellulolytic enzymes to degrade agricultural wastes into fermentable sugars, which requires lower temperature and produces relatively simple products [4].

Lignocellulose mainly consists of cellulose (30–45%), hemicellulose (15–30%) and lignin (12–25%), although the ratios may vary among feedstocks [5]. Therefore, industrial enzymatic degradation of lignocellulose highly depends on the synergism between xylanases, cellulases and lignin modification enzymes. Cellulose consists of β-1,4-linked glucosyl residues and is the most abundant component of lignocellulosic residues. The degradation of cellulose requires three types of cellulases for complete hydrolysis to glucose. Endoglucanases (EGs, EC 3.2.1.4) randomly cleave the internal bonds of amorphous cellulose molecules, exposing new accessible reducing and non-reducing ends. Exoglucanases (EXOs, EC 3.2.1.91) recognize these reducing and non-reducing ends and eventually cleave the cellulose polymers into trisaccharide cellotriose and disaccharide cellobiose. Cellobiose is further hydrolyzed by the action of β-glucosidases (BGLs, EC 3.2.1.21), producing glucose, which is readily usable for biofuel production [6]. Hemicellulose hydrolysis requires two additional types of enzymes, xylanases (EC 3.2.1.8) and β-xylosidases (EC 3.2.1.37), capable of degrading xylan into xylose [7]. The degradation of lignin requires the collaboration of laccases (EC 1.10.3.2) and class II peroxidases [8, 9].

Although the biochemical route requires lower energy input, the efficiency and stability of cellulases limits its use for long-term processing of agricultural waste. Furthermore, the cost of using cellulolytic enzymes to convert agricultural wastes into fermentable sugars is usually high [10]. To solve these problems, we need to discover an organism producing and secreting a high level of cellulases with good activity and stability. Most cellulases are produced by bacterial and fungal species, so researchers have been looking for highly efficient microbial cellulases to facilitate the cellulose decomposition process [11].

To achieve complete cellulose decomposition in industry, three types of cellulases have usually been used as a mixture from different fungal secretions because a single fungus is usually highly productive for only one or two types of cellulases [12, 13]. *Trichoderma reesei* is one of the most extensively used cellulase producers, especially for EXOs; however, its BGLs account for only ∼ 0.5% of the total secreted cellulases [14, 15]. Therefore, finding a novel organism that can produce and secrete all three types of cellulases would be beneficial. In addition to saprophytic fungi such as *T. reesei*, endophytic fungi have high potential for cellulase production and secretion. Recent studies of endophytes suggest that they produce volatile organic compounds applicable for biodiesel production, and the genomes of endophytic fungi encode a variety of carbohydrate-active enzymes (CAZymes) that may facilitate the degradation of lignocellulosic residues [16, 17]. Although genomic analyses have provided high-throughput annotations of genes encoding potential cellulolytic enzymes, the expression of their protein products and their enzymatic activity of the secreted cellulases need to be investigated.

To efficiently verify cellulolytic enzyme activities, researchers have used various artificial substrates to identify specific cellulase activities. Filter paper (FP) assays have been extensively used for determining total cellulase activities [18]. Avicel and 4-methylumbelliferyl-β-d-cellobioside (MUC) are commonly used as substrates for evaluating EXO activities [19]. 4-Methylumbelliferyl-β-d-glucopyranoside (MUD) and *p*-nitrophenyl β-d-glucopyranoside (*p*NPG) are used for assessing BGL activity [20, 21]. However, some substrates may be used by more than one type of cellulase; for instance, both EXO and BGL can hydrolyze MUC, and their activities cannot be distinguished in test tube assays [19, 22–24]. Zymography may be a good alternative for screening potential microorganisms with high cellulase activities. Crude protein extracts from microorganisms can be directly loaded onto native polyacrylamide electrophoresis (PAGE) gels and cellulase activities can be easily visualized by adding colorogenic or fluorogenic substrates during staining [25]. Although both EXOs and BGLs can hydrolyze MUC, previous studies indicated that BGL activity is repressed by its product glucose via competitive inhibition [24], but EXOs do not respond to glucose inhibition [26].

Taking advantage of this glucose product inhibition property of BGLs, we have established a simple zymography assay that can clearly distinguish EXO activities without interference from BGLs. We discovered that the *Daldinia caldariorum* strain D263 fungus secretes a wide range of cellulolytic and hemicellulolytic enzymes, including EXOs, EGs, BGLs and xylanases. Our D263 genome assembly and annotation indicated that D263 may encode more than 100 potential cellulolytic enzymes, and the proportion among different types of CAZymes in D263 is distinct from that in *T. reesei* and *A. niger*, which have been used for commercial cellulase production. We also successfully identified bioactive cellulases in the D263 secretion by coupling zymography and proteomic analyses. This study demonstrated that combining specific zymography and mass spectrometry analysis provides a convenient method for identifying cellulolytic enzymes. With this approach, the D263 fungus was determined as a good source of these enzymes for facilitating second-generation biofuel production.

## Results and discussion

### D263 produces and secretes a wide range of cellulolytic enzymes

**Fig. 1:**
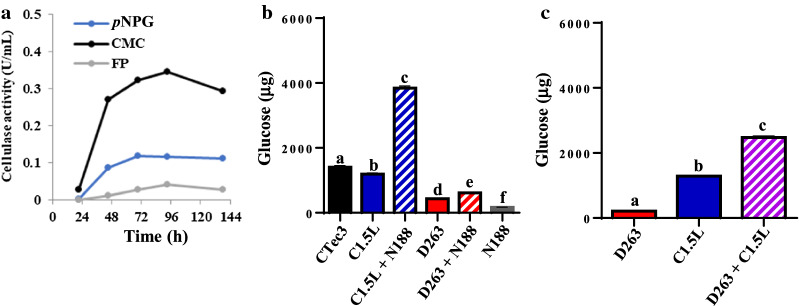
D263 is capable of degradation of cellulosic residues. **a** Filter paper (FP) and cellulase activities detected in crude D263 secretome. D263 was cultured in 1-L fermenter containing Mandel–Reese medium (pH 7) for 5 days. Data were shown as one representative of three biological replicates. All experiments showed similar results. **b** D263 is capable of hydrolyzing agricultural waste. The glucose generation from 10 mg sugarcane bagasse in 1 mL of reaction volume was used to evaluate the efficiency of the enzyme mixtures. Commercial enzymes CTec3, C1.5L and N188 were used as controls. For each assay, 0.14 FP unit (FPU) for CTec3, C1.5L or D263 was used in the presence or absence of 0.5 cellobiase unit (CBU) of N188 that acts as β-glucosidase supplement. Alphabets on the bars indicate significant difference in one-way ANOVA analysis with Fisher’s LSD (*p* < 0.05). **c** D263 acts synergistically with C1.5L. 0.05 FPU of D263 and 0.07 FPU of C1.5L were mixed or separately added to 10 mg of sugarcane bagasse in 1 mL of reaction. CMC, carboxymethylcellulose; *p*NPG*, p*-nitrophenyl β-d-glucopyranoside. Alphabets on the bars indicate significant difference in one-way ANOVA analysis with Fisher’s LSD (*p* < 0.05)

To discover fungal species suitable for producing and secreting a wide range of highly bioactive cellulolytic enzymes, we screened more than 40 strains of ascomycetes by examining the cellulolytic enzyme activities in their crude secretion. Among the fungal strains, we found several fungal species/strains with a variety of cellulosic activity (Table 1 and Additional file 1: Table S1). The crude secretion of *Chaetomella raphigera* D2 (D2) contained high BGL activity, as shown in our recent studies [27]. However, we found no EG, EXO or xylanase activity in the culture medium of D2. *Penicillium* sp. YS-40 also exhibited considerable BGL activity but low xylanase activity and no EXO and EG activity. *Fusarium proliferatum* showed high EG, BGL and xylanase activity and moderate EXO activity. *Aspergillus* sp. RS-19, *Neurospora* sp. RS-6 and *D. caldariorum* D263 could hydrolyze filter paper (FP), so these three species could produce several types of cellulolytic enzymes that can work synergistically for cellulose decomposition. EXO, EG and BGL activity was high in *Aspergillus* sp. RS-19, but no xylanase activity was detected in its secretion. *D. caldariorum* D263 also showed all types of cellulolytic enzymes (i.e., EG, EXO and BGL), with BGL activity higher than that in *Aspergillus* sp. RS-19 (1.82 vs 0.42 U/mL). D263 secretion also contained high xylanase activity (2.69 U/mL), which suggests that it has high potential for digesting hemicellulose. D263 showed relatively low activity on hydrolyzing the EXO substrate Avicel as compared with *Aspergillus* sp. (0.05 vs 0.18 U/mL), the commonly used fungal species for commercial enzymes. Hence, *D. caldariorum* D263 could be a good source for a complete set of cellulolytic enzymes or supplement of commercial cellulase because of its high BGL, EG and xylanase activity. We attempted to further mass-produce D263 cellulases in a 1-L fermenter. D263 crude secreted enzymes could hydrolyze carboxymethylcellulose (CMC) and *p*NPG, showing 0.29 and 0.11 U/mL activity, respectively, at 138 h. The D263 crude enzymes could also degrade FP, so they could completely digest crystalline cellulose (Fig. 1a).

**Table 1:** Cellulolytic enzyme activities of the examined ascomycete species

Fungal species	Cellulase activity (U/mL)				
	FP	Avicel	CMC	*p*NPG	Xylan
*Daldinia caldariorum* D263	0.38 ± 0.15	0.05 ± 0.03	0.73 ± 0.27	1.82 ± 0.90	2.69 ± 0.97
*Aspergillus* sp. RS-19	0.23 ± 0.03	0.18 ± 0.01	1.21 ± 0.03	0.42 ± 0.03	–
*Chaetomella raphigera* D2	–	–	–	0.98 ± 0.05	–
*Fusarium proliferatum*	–	0.25 ± 0.09	1.01 ± 0.10	1.43 ± 0.11	1.48 ± 0.64
*Neurospora* sp. RS-6	0.12 ± 0.01	0.13 ± 0.02	0.62 ± 0.16	0.13 ± 0.01	–
*Penicillium* sp. YS40-5	–	–	–	0.45 ± 0.09	0.05 ± 0.03

One unit of enzyme activity (U) was defined as 1 μmol of product generated per minute, FP, filter paper; CMC, carboxymethylcellulose; *p*NPG, *p*-nitrophenyl β-d-glucopyranoside

**Fig. 2:**
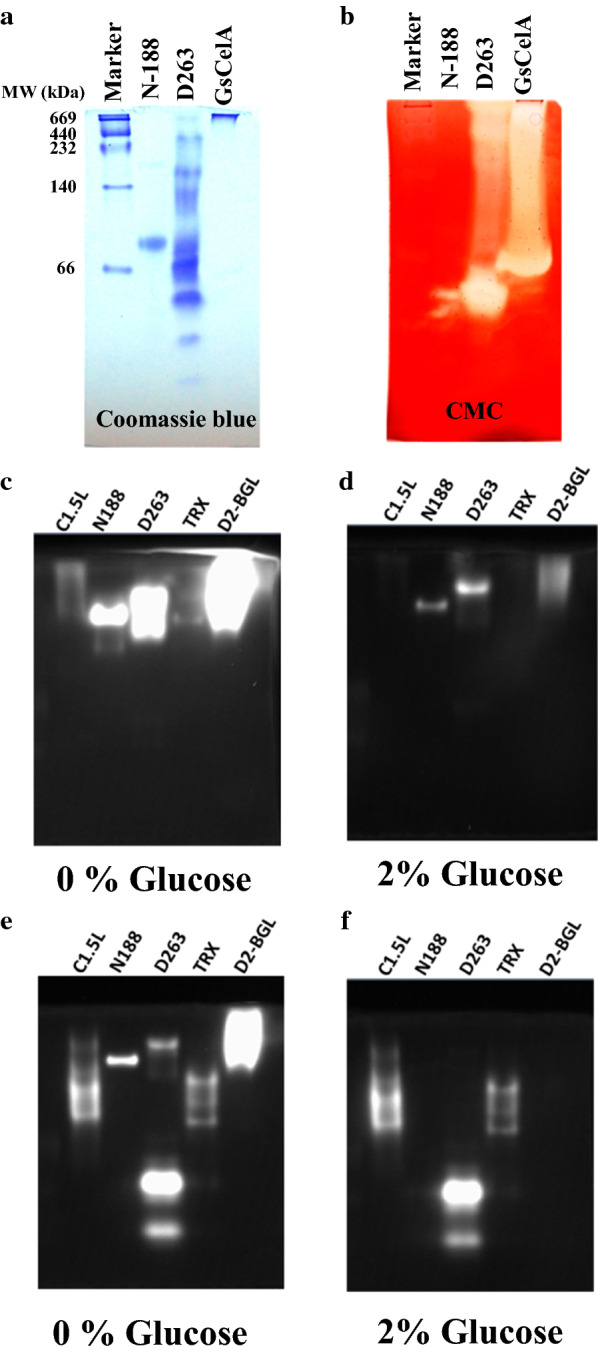
Addition of glucose helps specify the presence of exoglucanases on 4-methylumbelliferyl β-d-cellobioside (MUC) zymography. **a** Coomassie Blue staining of native PAGE for D263 crude enzyme. Novozyme 188 (N188) and *Geobacillus* sp. cellulase A (GsCelA) were negative and positive controls of endoglucanase activity, respectively. **b** D263 exhibits high endoglucanase activity. CMC zymography was used for detecting endoglucanase activity of the enzyme preparations. **c**, **d** D263 shows β-glucosidase activity. 4-Methylumbelliferyl-β-d-glucopyranoside (MUD) zymogram was used for detecting β-glucosidase activity. The addition of 2% glucose successfully represses β-glucosidase activity on the MUD zymography. Commercially available N188, Cellulast 1.5L (C1.5L), *Chaetomella raphigera* D2 β-glucosidase (D2-BGL) and laboratory-produced Trichoderma cellulase preparation (TRX) were used as positive and negative controls of glucose suppression of BGL activity. **e**, **f** D263 shows clear exoglucanase activity. MUC zymogram with 2% glucose addition was used for detecting exoglucanase activity of different cellulase preparations. The modified MUC zymography method can detect exoglucanase activity with little interference from β-glucosidase activity. The protein amount used in **a**–**e** was C1.5L, 2600 ng; N188, 150 ng; D263, 340 ng; TRX, 1100 ng; D2-BGL, 100 ng (for MUD assay) and 1000 ng (for MUC assay). All experiments were conducted on 8% native gel with at least three biological replicates

**Fig. 3:**
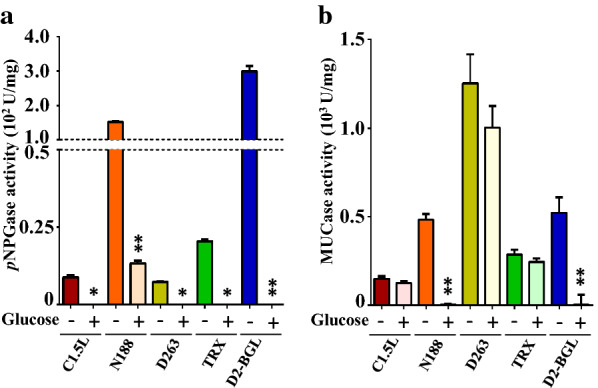
Glucose inhibits BGL but not exoglucanase activity in liquid assays. **a** *p*NPGase activity with different cellulase preparations. The enzyme preparations were reacted to *p*NPG in the presence or absence of 2% glucose. The specific activity (U/mg) was defined as 1 mmol glucose produced per min per 1 mg of crude enzyme from crude preparations of C1.5L, N188, D263, TRX and from purified D2-BGL, respectively. *p < 0.05, **p < 0.01 by Student’s *t*-test compared to their corresponding controls (without glucose). **b** MUCase activity with different cellulase preparations. The enzyme preparations were reacted with MUC in the presence or absence of 2% glucose. The specific activity (U/mg) was defined as 1 mmol 4-methylumbelliferone produced per min per 1 mg of crude enzyme from crude preparations of C1.5L, N188, D263, TRX and from purified D2-BGL, respectively. **p < 0.01 by Student’s *t*-test compared to their corresponding controls (without glucose)

To test whether D263 could hydrolyze agricultural waste into fermentable sugars, we incubated the crude enzymes of D263 with acid-pretreated sugarcane bagasse. Under the same FP unit, the secreted D263 cellulases could produce > 500 μg glucose from 10 mg sugarcane bagasse, although relatively lower than the commercial enzyme mixtures CTec3 and C1.5L (Fig. 1b). Interestingly, the mixture of D263 and C1.5L also showed a synergistic effect on decomposing sugarcane bagasse, (Fig. 1c). The commercial cellulase mixture C1.5L from *T. reesei* is high in EG and EXO activity, but is low in BGL activity [27], so the addition of D263 may act as a supplement of BGL activity that enhances the glucose production. In contrast, such synergism was not observed when mixing D263 secretion with CTec3 (Additional file 2: Fig. S1), consistent with previous reports that CTec3 contains high EG, EXO and BGL activity alone, so addition of D263 only has limited effect on promoting sugarcane bagasse decomposition. To further identify the bioactive cellulases in the D263, we used CMC zymography. D263 culture medium showed high EG activity (Fig. 2a, b). To distinguish EXO activities from BGLs, we developed an improved version of MUC zymography. In previous studies, it is known that the activity of BGLs is highly repressed by its product glucose [27–29]. Our previous research also showed that inhibition constants for glucose (i.e., *K*_*i*_ glucose) of D2-BGL and N188 were 2.42 mM and 4.68 mM, respectively [27]. Therefore, we included 2% of glucose (111.1 mM) in the zymography assay in order to identify EXO activity without the interference of BGL activity. As a proof of our concept, we first used MUD zymography to confirm BGL activity in bona fide BGL (i.e., N188 and D2-BGL because of their sensitivity to high levels of glucose on zymography). On MUD zymography, D263, N188 and D2-BGL showed strong BGL activity but C1.5L and TRX only weak BGL activity (Fig. 2c, d). A similar situation was observed with MUC as the substrate (Fig. 2e, f). We also used a liquid assay (i.e., *p*NPG assay) to confirm that all five cellulase preparations had BGL activity, which was greatly inhibited with 2% glucose (Fig. 3a). As described above, activity observed from N188 and D2-BGL in MUD zymogram was sharply decreased in the presence of 2% glucose, so they were indeed BGLs and act as positive controls of BGL activity in our zymography. We have also showed that cellulase preparations C1,5L and TRX with expected high EXO activity but low BGL activity could serve as negative controls in our MUC zymography, since both of them showed similar signal intensities with and without glucose in MUC zymogram (Fig. 2e, f). Consistent with our zymography results, MUC liquid assay revealed that the MUCase activity was mostly derived from BGL activity in N188 and D2-BGL, which was severely reduced with the addition of glucose, while only a slight decrease was observed in C1.5L, D263 and TRX (Fig. 3b).

In addition to containing BGL activity, D263 cellulase preparation contained EXO activity, revealed on the MUC zymogram as bands that were insensitive to 2% glucose (Fig. 2e, f). Therefore, BGL activity was highly repressed by the high concentration of glucose on both MUD and MUC zymograms. We applied this concept to reveal EXO activity with MUC zymography. For BGLs such as N188 and D2-BGL, signals in the MUD zymogram were also observed in the MUC zymogram because of the ability of BGL to also hydrolyze MUC (Fig. 2e). However, in the MUC zymogram, other positive signals were observed for cellulase preparations D263, C1.5L and TRX because of the presence of EXOs. In the presence of 2% glucose, the MUC-positive signals in D2-BGL, N188 and the high-molecular-weight band of D263 were greatly diminished, whereas those of C1.5L, TRX and low-molecular-weight band of D263 remained largely unchanged (Fig. 2e, f). The remaining MUC-positive signals in the presence of 2% glucose strongly suggest that EXO activity in the fungal cellulase extracts is not sensitive to glucose inhibition (Fig. 2f). The results also demonstrate that most of the MUCase activity from D263, TRX and C1.5L resulted from EXOs. Recent reports suggested that some of the cellulase activity can be readily detected on denaturing gel zymograms [29]. Many hydrolytic enzymes can be denatured by the ionic detergent SDS, but renatured later if SDS is removed, which results in a zymographic technique allowing for the separation and detection of specific enzymes by SDS-PAGE with better resolution.

**Fig. 4:**
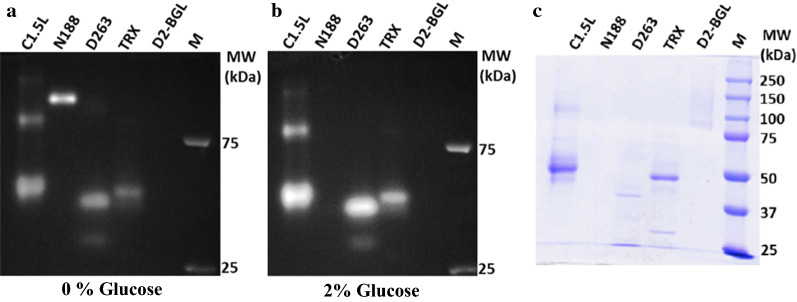
SDS-PAGE can be used for detecting exoglucanase activity on MUC zymography. **a** MUC zymography can also reveal cellulase activities on SDS-PAGE gels. **b** Addition of 2% glucose in MUC zymography represses BGL activity on SDS-PAGE gel. N188, C1.5L, TRX and D2-BGL were used as positive and negative controls of glucose suppression of BGL activity. **c** Coomassie Blue staining shows protein samples loaded into SDS-PAGE gel. The protein amount used was C1.5L, 2600 ng; N188, 150 ng; D263, 340 ng; TRX, 1100 ng; D2-BGL, 1000 ng. All experiments were conducted on 8% SDS-PAGE gels with at least three biological replicates

Therefore, we also examined the MUC zymogram by using SDS-PAGE to give a more precise estimation of protein molecular weight. The EXO activity in the MUC zymogram was only slightly affected by SDS (Fig. 4a–c). We still observed the glucose repression of BGL activity, and D2-BGL activity could not be detected on SDS-PAGE zymograms (Fig. 4a, b). This observation is consistent with our previous findings that *Pichia pastoris*-produced recombinant D2-BGL activity was sensitive to SDS and could not be renatured even after SDS was removed [30]. This finding is likely to due to unusual glycosylation of this enzyme in *Pichia* [30].

In this work, we have devised a simple, yet specific, zymographic detection of EXO secreted from the fungus *D. caldariorum* D263. With our improved MUC zymography method, we successfully demonstrated the presence of EGs, EXOs and BGLs from the D263-secreted cellulases, so D263 may produce a wide range of cellulolytic enzymes capable of decomposing hemicellulose and cellulose, which are not commonly available in commercial cellulase mixtures such as C1.5L (primarily from *T. reesei*) and N188 (primarily from *A. niger*).

### D263 genome encodes lignocellulolytic enzymes distinct from commercially used fungi

**Fig. 5:**
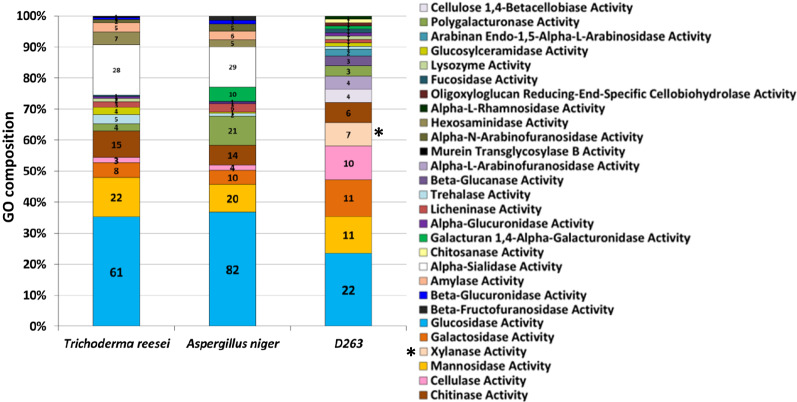
Gene Ontology (GO) revealed specific enrichment of potential cellulases in the D263 and ascomycete genomes. Potential lignocellulolytic enzymes in the assembled D263 genome and that of *Trichoderma reesei* and *Aspergillus niger* were annotated by GO analysis (GO depth ≥ 6). Numbers within the bars indicate genes belong to the specific GO terms. Asterisk indicate GO terms specifically enriched in D263

**Fig. 6:**
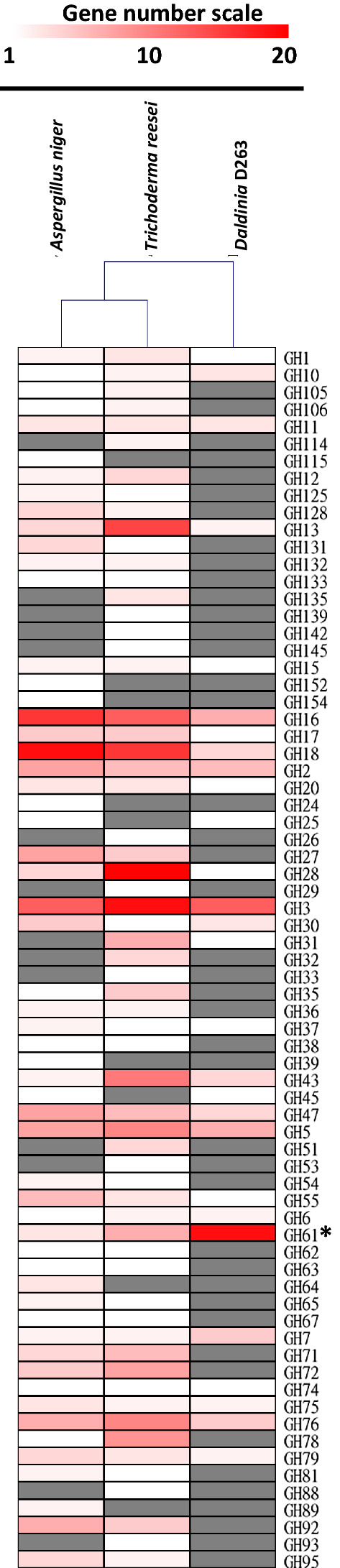
D263 CAZymes are distinct from those in *Aspergillus niger* and *Trichoderma reesei*. Heat map showing the distribution of glycoside hydrolase (GH) family proteins in D263, *A. niger* and *T.* *reesei* genomes, respectively. White-to-red color scale reflects the number of GH family proteins, gray color indicates the GH family protein was not present in the corresponding fungal genome. Hierarchical clustering based on Pearson correlation between fungal species shows that the GH family distribution between *A. niger* and *T. reesei* are more similar than to D263. Asterisk indicates highly presented GH family in D263 genome

From our zymography and liquid assays, D263 was able to degrade both cellulose and hemicellulose (Table 1, Figs. 1, 2). To identify highly bioactive cellulases that have high potential for industrial application, it is necessary to further purify the enzymes of interest from the D263-secreted cellulolytic enzymes. However, the purification and identification process can be laborious without knowledge of the D263 genome. To acquire sufficient genomic information, we have taken advantage of DNA sequencing technology to assemble the draft genome of D263. We obtained 195 contigs from our 454 sequencing, which could be further assembled into 110 scaffolds. The assembled genome size of D263 is 37.8 Mbp, with 10,477 genes annotated and a gene density of 277.4 genes/Mbp (Table 2). The genome size and gene density resemble those of recently published endophytic fungal genomes. We have also calculated the GC content of D263 and previously reported endophyte genomes from Mycocosm database [31]. The GC content between D263 and *Daldinia* EC12 were quite similar and were slightly higher than the three strains of *Hypoxylon* spp. published in previous literature [17]. To further investigate the genes in D263 genome with potential for cellulose degradation, we have conducted Gene Ontology (GO) analysis to annotate the potential functions of D263 genes. The BLAST2GO tool enables us to decipher the functions of the potential genes based on their sequence similarities to the closest fungal species [32]. Our analysis revealed that around 10% of genes in the D263 genome (1046 genes) may encode hydrolases (Additional file 3: Table S2). Most of the lignocellulolytic enzymes are hydrolases, including glycoside hydrolases (GHs) that are essential for the degradation of various lignocellulosic residues. Because we observed that D263 produces a wide range of EG, EXO, BGL and xylanase activity, we compared the GO term distribution for D263 and the commercially used fungi *T. reesei* and *A. niger* (Fig. 5). All three fungi were enriched in glucosidases and mannosidases. The former is responsible for catabolizing complex carbohydrates into simple sugars, while the latter was essential for the formation of fungal cell wall. Interestingly, the D263 genome also showed specific enrichment of xylanases, which corresponds to the high xylanase activity observed in D263 secretion (Table 1). It is known that xylan is the most abundant non-cellulosic polysaccharides in plant cell wall, so the enrichment of xylanase genes and activity in D263 may provide efficient degradation of hemicellulose into five-carbon sugar xylose. Of note, both *T. reesei* and *A. niger* showed enrichment of sialidases, which were lacking in the D263 genome (Fig. 5). Sialidases are a group of GHs required for pathogen virulence. In microorganisms, sialidases function to hydrolyze sialic acid for nutritional purposes and are involved in adhesion and host cell infection [33]. Also, an *Aspergillus fumigatus* sialidase (Kdnase) may be responsible for its virulence and cell wall integrity [34]. However, sialic acid is barely present in plants [35, 36], thus sialidases may not be needed for cellulose decomposition. To further identify the D263-specific cellulolytic enzymes, we have also compared the amino sequences of potential D263 *O*-glycosyl hydrolases in the Carbohydrate-Active enZYme (CAZy) database. GHs are widespread enzyme groups that hydrolyze glycosidic bonds rich in cellulose and hemicellulose [16, 37–39]. We found that the D263 genome encodes at least 103 GH proteins. To further validate the distinct features of D263 cellulolytic enzymes, we have compared the GH distribution of D263 to the commercial cellulase producing fungi *T. reesei* and *A. niger* (Fig. 6).

**Table 2:** Genome assembly comparisons of D263 and related endophyte species

	*Daldinia* D263	*Daldinia* EC12 [17]	*Hypoxylon* CI4A [17]	*Hypoxylon* CO27 [17]	*Hypoxylon* EC38 [17]
Genome size (Mbp)	37.8	37.5	37.7	46.5	47.3
GC content (%)	44.7	44.9	45.8	40.5	39.8
Contigs	195	641	1044	505	1168
Number of genes	10,477	11,173	11,712	12,256	12,261
Gene density (genes/Mbp)	277.4	298	311	263	259
Exon frequency (exon/gene)	2.87	2.89	2.84	2.9	2.86

The GC content of the fungal genome from *Wu *et al*.* were calculated based on their uploaded genomes in the Mycocosm database

All three fungi encode a large number of GH3, GH5 and GH16 enzymes. GH3 family proteins have a wide range of functions in cellulosic biomass degradation, including BGL and β-d-xylopyranosidase activity [40, 41]. Although *T. reesei* contained more GH3 proteins compared to *A. niger* and D263, its BGL yield is the lowest among the three fungal species [27]. The results were supported by previous literature that *T. reesei* strain RUT-C30 cannot efficiently secrete BGLs to its environment, so low BGL activity was detected in its secreted enzymes [42]. GH5, on the other hand, is one of the important protein families with high EG and xylanase activity [43]. The GH16 family contains various β-galactanases and xyloglucan transglucosylases/hydrolases (XTHs) that may be required for xylan decomposition [16, 44, 45]. The enrichment of GH5 and GH16 family proteins is consistent with their high EG and xylanase activity in our observation (Fig. 1, Table 1). Interestingly, there are GH proteins that were specifically enriched in each fungal species.

The most presented members of GH family proteins are GH18 in *T. reesei* and GH28 in *A. niger* (Fig. 6). GH18 family proteins are enriched in chitinases, which were able to degrade the fungal cell wall and are essential for the mycoparasitic activity in *Trichoderma* spp. fungi [46]. *A. niger* produces GH28 polygalacturonases that belong to pectinases that were required for its virulence against plant tissues and were widely used in the food industry [47]. These two families were not enriched in D263, which is consistent with previous study that no endophytes tested showed pectinase activity and endophytes were usually considered as latent pathogens [48].

The GH61 family proteins, on the other hand, are most abundant in the D263 genome, but only a few existed in *Aspergillus* and *Trichoderma* genomes. GH61 enzymes are classified as auxiliary activity family 9 (AA9) lytic polysaccharide monooxygenases (LPMOs) and are involved in the cleavage of (1,4)-linked glycosidic bonds on the surface of crystalline plant polysaccharides, hence increasing substrate accessibility of other cellulases [49]. Also, co-expression of a LPMO in a commercial strain (i.e., *T. reesei*) enhanced the conversion of plant biomass [50]. A recent study showed that LPMOs may act synergistically with xylobiohydrolase to facilitate the degradation of xylans [51]. This specific enrichment of GH61 proteins in D263 may also facilitate the activity of its EG and xylanase activity, resulting in high activity observed in D263 secretion (Table 1). Prompted by this notion, we have assayed the activity of GH61 proteins in D263 cellulase preparation. In recent study, it was suggested that LPMO activity may be visualized on CMC zymography with the use of a reducing agent such as ascorbic acid [52]. However, our results indicated that no additional band was observed in the presence of 10 mM ascorbic acid on both native and SDS-PAGE gels (Additional file 2: Fig. S2A–D). Thus, the probable LPMO activity in the gel could be masked by the high EG activity of D263. Our result also suggests that the activity of LPMOs may be significantly lower than that of EGs, and both types of enzymes showed similar molecular weight on zymography. Taken together, our genomic analyses suggest that D263 may encode a group of plant biomass-degrading enzymes that are distinct from *A. niger* and *T. reesei,* which makes this organism a novel source of cellulosic enzymes with potential of producing novel AA9 LPMOs useful for the biofuel industry.

### Validation of cellulolytic enzymes secreted by D263

**Fig. 7:**
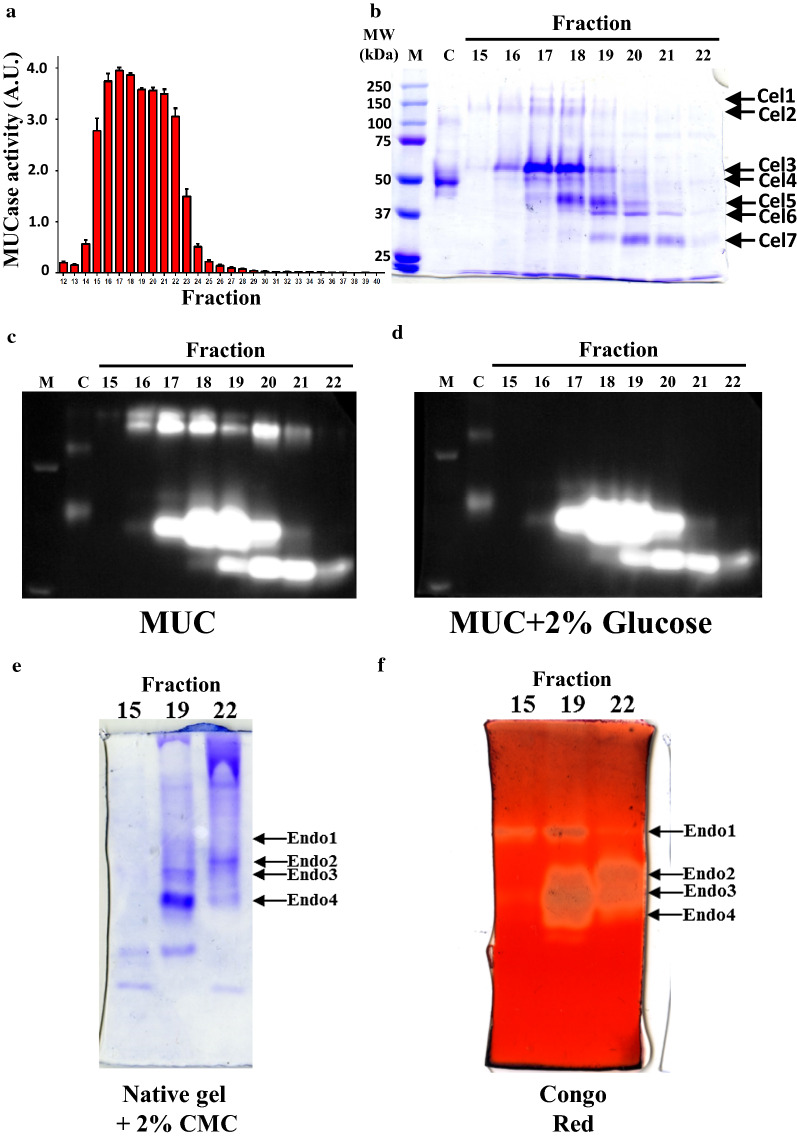
Cellulases with confirmed activities or peptide sequences determined by mass spectrometry. **a** MUCase activities of D263 elution profile after purification by ion-exchange chromatography. The unit of MUCase activity was calculated as changes in OD_450_ per minute. **b** Coomassie Blue staining showing the purified fractions with high MUCase activities on 8% SDS-PAGE gel. The seven major bands on the gel were indicated as Cel1–Cel7. M: prestained protein marker; C: C1.5L that served as positive control MUCase activity on the zymography. **c** MUC zymography of the purified D263 fractions with high MUCase activity in the absence of glucose. **d** Visualization of exoglucanase activity in the purified D263 fractions. The MUC zymography was conducted in the presence of 2% glucose. **e** Coomassie Blue staining showing purified D263 fractions on native PAGE gel containing 2% of carboxylmethylcellulose (CMC). The four major bands labeled as Endo1–Endo4 were potential endoglucanase candidates. **f** Congo red zymography shows high endoglucanase activities in the purified D263 protein fractions. Highly MUC-positive signals were present at the corresponding regions of Endo1–Endo4

In addition to the discovery of new LPMO genes, our GO analyses revealed that the D263 genome may encode more than 50 cellulolytic enzymes, including at least 12 EGs, 4 EXOs, 9 BGLs, 19 LPMOs and 7 xylanases (Additional file 3: Table S2). The D263 EXOs belong to GH6 and GH7 families. Many members of these two families are known as secretory cellulose 1,4-β-cellobiosidases [53]. Although D263 has shown lower capability of Avicel degradation than *Trichoderma* and *Aspergillus* (Table 1), it indeed showed strong MUCase activity (Figs. 2f, 3b). Amino acid sequences of protein bands identified from zymograms of enzymatic activity can be determined by mass spectrometry, then used for annotation of genomic sequences encoding this enzyme. To identify the active cellulases secreted by D263, we partially purified the crude enzyme of D263 by using anion-exchange chromatography and detected MUC activities in each elution fraction. The fractions 15 to 22 contained proteins enriched with MUCase (Fig. 7a). We demonstrated high BGL and EXO activities on MUC zymograms corresponding to seven protein bands (Cel1–Cel7) on Coomassie Blue-stained gel (Fig. 7b–d). Our proteomic analyses indicated that the major proteins of Cel1 band were likely GH3 BGLs (genes 006152 and 001615), consistent with the observation that Cel1 showed obvious glucose inhibition during MUC zymography analysis (Fig. 7c, d, Table 3 and Additional file 4: Table S3). The most enriched protein in Cel2 was 1,4-β-d-xylosidase (gene 005328), consistent with our observation that D263 was enriched in xylanases (Table1, Fig. 5). However, although some of the peptides belong to Cel1 BGLs can also be identified in Cel2 (Table 2, Fig. 7 and Additional file 4: Table S3). The shift of molecular weight observed in the putative 1-4-β-d-xylosidase 005328 may be resulted from post-translational modification such as glycosylation. The enrichment of putative enzyme 005328 also supported the discovery that D263 showed enrichment of xylanases (Fig. 5). Cel3 to Cel5, on the other hand, correspond to EXOs of 42 to 49 kDa (genes 006394, 007734 and 010048), which clearly reflects to the MUCase positive signals in the MUC zymography with 2% glucose (Fig. 7c, d, Table 3 and Additional file 4: Table S3). Interestingly, the enriched fractions 19 and 22 also showed high EG activity in these molecular-weight regions, with one EG identified as the best matched protein of Cel6 (gene 002528) (Fig. 7e, f, Table 3, Additional file 4: Table S3 and Additional file 5: Table S4). Surprisingly, Cel7 was identified as only a partial fragment of endo-1,4-β-xylanase (gene 004812). It is known that the hydrolysis of xylan requires the orchestrated actions of endoxylanases and the β-xylosidases [54]. The identification of both 1,4-β-d-xylosidase (gene 005328) and endo-1,4-β-xylanase (gene 004812) suggests that D263 is capable of complete hydrolysis of xylan. Further investigation of D263 xylanases may be beneficial for discovering new and highly efficient xylan decomposition enzymes that can be used in industry. Interestingly, the identified cellulolytic enzyme may be observed from multiple major bands of both native and SDS-PAGE gels after our proteomic analysis, with their molecular weight slightly shifted from the molecular weight prediction based on amino acid sequences (Additional file 4: Table S3 and Additional file 5: Table S4). The differences may have resulted from post-translational modification of these cellulolytic enzymes, such as *N*-glycosylation and *O*-glycosylation. This phenomenon has been observed in many cases. Previous studies have shown that the *C. raphigera* expressed native D2-BGL has two glycosylation variants and the more extensively *O*-glycosylated form exhibited increased activity toward its natural substrate cellobiose [30]. The recombinant *A. aculeatus* *Aa*BGL1, a 91-kDa β-glucosidase with 16 potential *N*-glycosidation sites, showed a smeared band between 135 and 140 kDa during SDS-PAGE analysis [55]. It was also reported that *N*-glycosylation is critical for the folding of *Aspergillus terreus* BGL when heterologously expressed in *Pichia pastoris* and *Trichoderma reesei* [56]. Future studies on the glycosylation variants of D263 cellulolytic enzymes will aid us in identification of critical glycosylation sites that are essential for cellulolytic enzymatic activity. From our identification of active EGs in the Endo1–Endo4 protein bands, we have found that both Endo1 and Endo3 were enriched in a potential EXO, gene 010048, which was also enriched in Cel4 and Cel5 in our SDS-PAGE zymography. Endo2 was enriched in gene 006394, which was identified as a potential EXO that is abundant in Cel3 (Fig. 7b, f, Table 3). Endo 4 contains a GH7 EG (gene 002528) that was also identified in Cel6 of our SDS-PAGE zymography. The results suggested that these EXOs and EGs were abundant in this molecular weight region. Since the mobility on a native gel is determined by both size and charge of a protein, it is plausible that a large protein with positive charge could migrate to a similar position as a smaller protein with less positive charge, which may also explain the slight size difference between proteomic analyses and native PAGE results. Interestingly, the proteins identified in Endo4 also include a GH30 endo-β-1-6 glucanase (gene 009220) and a potential GH61 LPMO (gene 001982). LPMOs exist in a relatively low amount as compared with most cellulolytic enzymes identified in the protein fractions and are not easily visible on zymography (Table 3, Additional file 2: Fig. S2, Additional file 4: Table S3 and Additional file 5: Table S4). Our proteomic analysis clearly indicates that the endophytic fungus D263 can produce and secrete a large variety of bioactive cellulases (Table 3). By conducting RNA sequencing analysis using our gene annotation for mapping, we will be able to identify the optimal conditions for induction of D263 cellulolytic enzymes, which will facilitate cellulose degradation and second-generation biofuel production.

**Table 3:** List of D263 lignocellulolytic enzymes identified by mass spectrometry

Protein location	Gene	Enzyme type	GH family	Predicted MW (kDa)	Coverage (%)	Abundance	Mascot score
Cel1	gene001615	β-Glucosidase	GH3	94.9	8	47,659,991.44	591
Cel2	gene005328	1,4-β-d-Xylosidase	GH3	83.7	19	104,471,229	1222
Cel3	gene006394	Exo-glucanase	GH7	49.4	68	7,725,730,124	5414
Cel4/Cel5	gene010048	Exo-glucanase	GH7	48.1	13	59,128,446.88	560
Cel6	gene002528	Endo-glucanase	GH7	44.8	23	451,563,228.5	791
Cel7	gene004812	Endo-1,4-β-xylanase	GH7	38.7	20	27,919,145.25	1026
Endo1/Endo3	gene010048	Exo-glucanase	GH7	48.1	21	97,686,007.72	1410
Endo2	gene006394	Exo-glucanase	GH7	49.4	9	15,179,733.59	565
Endo4	gene002528	Endo-glucanase	GH7	44.8	49	3,121,800,591	3186

Lignocellulolytic enzymes with the highest Mascot score in each sample were shown

## Conclusions

The combination of zymography and mass spectrometry analyses provides a powerful tool for genome annotation and discovery of new cellulolytic enzymes usable in agricultural waste disposal. Here, we have discovered that the endophytic fungus *D. caldariorum* D263 is able to produce a wide range of cellulolytic enzymes that efficiently hydrolyzed acid-pretreated sugarcane bagasse. Our zymography revealed the presence of EXOs, EGs and BGLs in the secreted cellulolytic enzymes of D263. Addition of 2% glucose was essential for specific identification of EXOs without the interference of BGL activity in MUC zymogram. We have further identified the specific cellulolytic enzymes in D263 by integrative genomic and proteomic analyses. Our results also suggest that the AA9 LPMOs are more abundant in *D. caldariorum* D263 than that of commercially used fungi such as *T. reesei* and *A. niger*. Taken together, our research pipeline successfully proved that D263 has high potential for expressing and secreting a wide range of cellulolytic enzymes.

## Methods

### Chemicals and enzymes

Carboxymethylcellulose (CMC), 4-methylumbelliferyl-β-d-cellobioside (MUC), 4-methylumbelliferyl-β-d-glucopyranoside (MUD), *p*NPG and birchwood xylan were from Sigma-Aldrich (USA). Avicel PH-101 was from Fluka (USA). Acrylamide, ammonium persulfate and TEMED were from Bio-Rad (USA). The *A. niger* β-glucosidase Novozyme 188 (N188) was from Sigma-Aldrich (USA). The recombinant β-glucosidase *Chaetomella raphigera *D2-BGL was produced by our laboratory and purified by using immobilized metal affinity chromatography [27]. The commercial cellulase preparation Celluclast 1.5L (C1.5L) was from Sigma-Aldrich (USA), and the laboratory-made *T. reesei* cellulase preparation TRX was provided by the Institute of Nuclear Energy Research (Taoyuan, Taiwan).

### Fungal culturing conditions

Fungal mycelia were cultured on potato-dextrose agar (PDA; Difco, BD, USA) for 3 days. Ten discs (0.8 cm in diameter) of mycelia were transferred to a 50-mL modified Mandel–Reese (MR) medium [57] containing 1.4 g/L (NH_4_)_2_SO_4_, 2 g/L KH_2_PO_4_, 0.34 g/L CaCl_2_, 0.3 g/L MgSO_4_·7H_2_O, 5 mg/L FeSO_4_·7H_2_O, 1.6 mg/L MnSO_4_·7H_2_O, 1.4 mg/L ZnSO_4_·7H_2_O, 2 mg/L and CoCl_2_·6H_2_O in a 250-mL flask. Depending on the fungal strain cultured, the MR medium was supplemented with cellobiose, α-cellulose, filter paper or dried Napier grass as the carbon source, with 1 g/L soy peptone and 0.3 g/L urea as the nitrogen source. Cellulase and xylanase activities were assayed by using Whatman #1 filter paper, Avicel, CMC, *p*NPG and xylan for total cellulase, exoglucanase, endoglucanase, β-glucosidase and xylanase activities, respectively. For 1-L fermenter assay, 20 fungal discs (0.8 cm in diameter) were added to a 1-L benchtop fermenter (Firstek Scientific, Taiwan) containing 700 mL MR medium and 0.5% α-cellulose and cultured at 30 °C, 800 rpm.

### Purification of D263-secreted cellulolytic enzymes

The supernatant of D263 culture was filtered through a 0.45-μm filter, and the buffer exchange was performed with the binding buffer (50 mM Tris, pH 8) by using Vivaspin 20 (30 kDa MWCO, General Electric, USA). The anion-exchange chromatography involved using the ÄKTA-FPLC System (General Electric, USA) and a 5-mL HiTrap Q column (General Electric, USA). Elution was conducted with a linear gradient from 0 to 100% of elution buffer (20 mM sodium phosphate, 1 M NaCl, pH 8). The eluted fractions with cellulase activity were separated on SDS-PAGE gels for evaluation of MUCase activity and identification of major cellulolytic enzymes by mass spectrometry.

### Gel electrophoresis and zymography assays

Electrophoresis in 8% native or SDS polyacrylamide gels was conducted at 100 V for 30 min, then 120 V for 80 min, with the electrophoresis tank (Bio-Rad, USA) and placed on ice to preserve enzymatic activities. Protein samples were mixed with a 5× SDS-PAGE sample buffer (0.5 M Tris–HCl pH 6.8, 10% SDS, 50% glycerol, 5% β-mercaptoethanol, 0.05% bromophenol blue), or 5× native PAGE sample buffer (0.5 M Tris–HCl pH 6.8, 50% glycerol, 0.05% bromophenol blue). Protein samples were not boiled before loading in order to preserve cellulase activities. After electrophoresis, each gel was incubated with 20 mL of 50 mM sodium acetate (pH 5) for 30 min with gentle shaking for equilibration. MUC and MUD were used as substrates to detect EXO and BGL activities, respectively, in zymography assays. In brief, each gel was submerged in 20 mL of 0.5 mM MUC or 0.5 mM MUD prepared with or without 2% (w/v) glucose in 50 mM sodium acetate, pH 5. The submerged gels were incubated at 50 °C for 10 min, then washed three times with 20 mL of 50 mM sodium acetate, pH 5. EXO and BGL activities were visualized by fluorescence under UV-B light. Commercial cellulases N188 and C1.5L were used as positive controls for MUC and MUD assays, respectively. To detect EG activity, 0.2% CMC was incorporated into the SDS-PAGE gel. After the electrophoresis, the gel was incubated in the renature buffer (1% Triton X-100 and 10 mM Tris–HCl, pH 7) at 4 °C overnight. After washing with ddH_2_O, the gel was submerged into 50 mM sodium acetate buffer, pH 5 and incubated at 50 °C for 30 min. The reaction was stopped by adding 0.1 M Tris–HCl (pH 8) for 20 min, and the enzyme activity was revealed by staining with 0.2% Congo Red (Sigma-Aldrich, USA). For LPMO detection, we have followed previously published literature with slight modifications [52]. In brief, 8% of native or SDS-PAGE gels containing 0.2% of CMC were soaked in 50 mM sodium acetate buffer, pH 5, with or without 10 mM ascorbic acid. The soaked gels were incubated at 50 °C for 16 h and the enzyme activity was revealed by staining with 0.2% Congo Red (Sigma-Aldrich, USA).

### Cellulase activity assays in test tubes

For BGL activity assays, 100 μL of enzyme solution was mixed with 100 μL of 4 mM *p*NPG in 50 mM sodium acetate buffer, pH 5, with or without 2% (w/v) glucose. The reaction was performed at 55 °C for 5 min under agitation and was stopped by adding 600 μL of 1 M Na_2_CO_3_. The product concentration was determined by measuring the OD_405_ of the final reaction solution with a BioTek GEN5 spectrophotometer. Serially diluted 4-nitrophenol solutions were used to establish the standard curve. For EXO activity assays, 100 μL of 1 mM MUC with or without 2% (w/v) glucose was mixed with 100 μL enzyme solution. The fluorescence was detected with a BioTek GEN5 spectrophotometer with excitation and emission wavelengths set at 360 and 450 nm, respectively. Serially diluted 4-methylumbelliferone was used for establishing the standard curve.

### Genomic DNA isolation

*Daldinia caldariorum* D263 was cultured on PDA plate for 3 days, and the mycelium discs were transferred to MR medium with 0.5% α-cellulose and incubated for 5 days. The fungal mycelia were washed with distilled water and then ground into fine powder in liquid nitrogen. To extract genomic DNA, 12 mL urea extraction buffer (7 M urea, 0.3 M NaCl, 50 mM Tris–Cl, pH 8, 20 mM EDTA, 1% sarcosine, pH 8) was added to the ground mycelia and mixed by vortexing. A 12-mL amount of phenol:chloroform:isoamyl alcohol (25:24:1) was then added and mixed for 15 min at room temperature. The sample was then centrifuged for 10 min at 8000 rpm, 4 °C. The supernatant was transferred to a clean tube and mixed with 2 mL of 4.4 M ammonium acetate and 13 mL isopropanol. After centrifugation for 10 min at 8000 rpm, 4 °C, the supernatant was discarded and the pellet was washed twice with 1 mL of 100% EtOH. The pellet was air-dried and re-suspended in 200 μL ddH_2_O and heated for 1 h at 65 °C. After heating, 20 μL RNase A (10 mg/mL) was added and the sample was incubated at 37 °C for 30 min. After incubation, the sample volume was adjusted to 500 μL with ddH_2_O.

### Genome sequencing and bioinformatic/proteomic analyses

About 400 μg isolated genomic DNA was used for library preparation, and 454 sequencing (Roche, Switzerland) was used to acquire sequence reads with average length of 373 bp. The draft genome of D263 was assembled by using Newbler V2.3, with mapping statistics shown in Additional file 1: Table S1. Blast2Go was used to identify enriched Gene Ontology (GO) terms in D263 genes [58]. GOBU was used to characterize the GO terms in the D263 genome [59]. KofamScan was used to predict the KEGG pathways of D263 genes [60]. The mapping statistics are shown in Additional file 6: Table S5. This Whole Genome Shotgun project has been deposited at DDBJ/ENA/GenBank (Accession no. JAAOZU000000000). The GO terms and CAZy enzymes in *T. reesei* V2.0 and *A. niger* ATCC1015 were searched in the Joint Genome Institute (JGI) MycoCosm database [31, 61–63]. Hierarchical analysis was conducted based on Pearson correlation. For proteome analyses, protein bands showing cellulolytic enzyme activities were extracted from SDS-PAGE gels. The gel slices were trypsin-digested and underwent mass spectrometry. The amino acid sequences of annotated D263 genes underwent a Mascot search (Matrix Science, USA). Protein abundance was calculated by using ProteomeDiscoverer v2.4 (Thermo-Fisher, USA).

## Supplementary Information

**Additional file 1: Table S1.** Optimal growth conditions for the ascomycetes tested.

**Additional file 2: Figure S1.** D263 does not supplement the activity of CTec3. **Figure S2.** No D263 LPMO activity visualized on zymography.

**Additional file 3: Table S2.** GO categorization of annotated D263 genes.

**Additional file 4: Table S3.** Identified D263 proteins from the MUCase activity bands Cel1–Cel7.

**Additional file 5: Table S4.** Identified D263 proteins from the CMCase activity bands Endo1–Endo4.

**Additional file 6: Table S5.** Mapping statistics of D263 genome.

## Data Availability

This Whole Genome Shotgun project has been deposited at DDBJ/ENA/GenBank (Accession no. JAAOZU000000000). The version described in this paper is JAAOZU010000000.

## Abbreviations

BGL: β-Glucosidase
CAZymes: Carbohydrate-active enzymes
EG: Endoglucanase
EXO: Exoglucanase
FP: Filter paper
GO: Gene Ontology
LPMO: Lytic polysaccharide monooxygenase
MUC: 4-Methylumbelliferyl-β-d-cellobioside
MUD: 4-Methylumbelliferyl-β-d-glucopyranoside
PAGE: Polyacrylamide gel electrophoresis
*p*NPG: *p*-Nitrophenyl β-d-glucopyranoside
