# Supplementary material for: From simple and specific zymographic detections to the annotation of a fungus Daldinia caldariorum D263 that encodes a wide range of highly bioactive cellulolytic enzymes
Source: Biotechnol Biofuels. 2021 May 21;14:120. doi: 10.1186/s13068-021-01959-1 (PMC8140500; doi:10.1186/s13068-021-01959-1)
Supplement: Supplementary file 1 — Additional file 1: Table S1. Optimal growth conditions for the ascomycetes tested. [file 13068_2021_1959_MOESM1_ESM.pptx]

## Slide 1
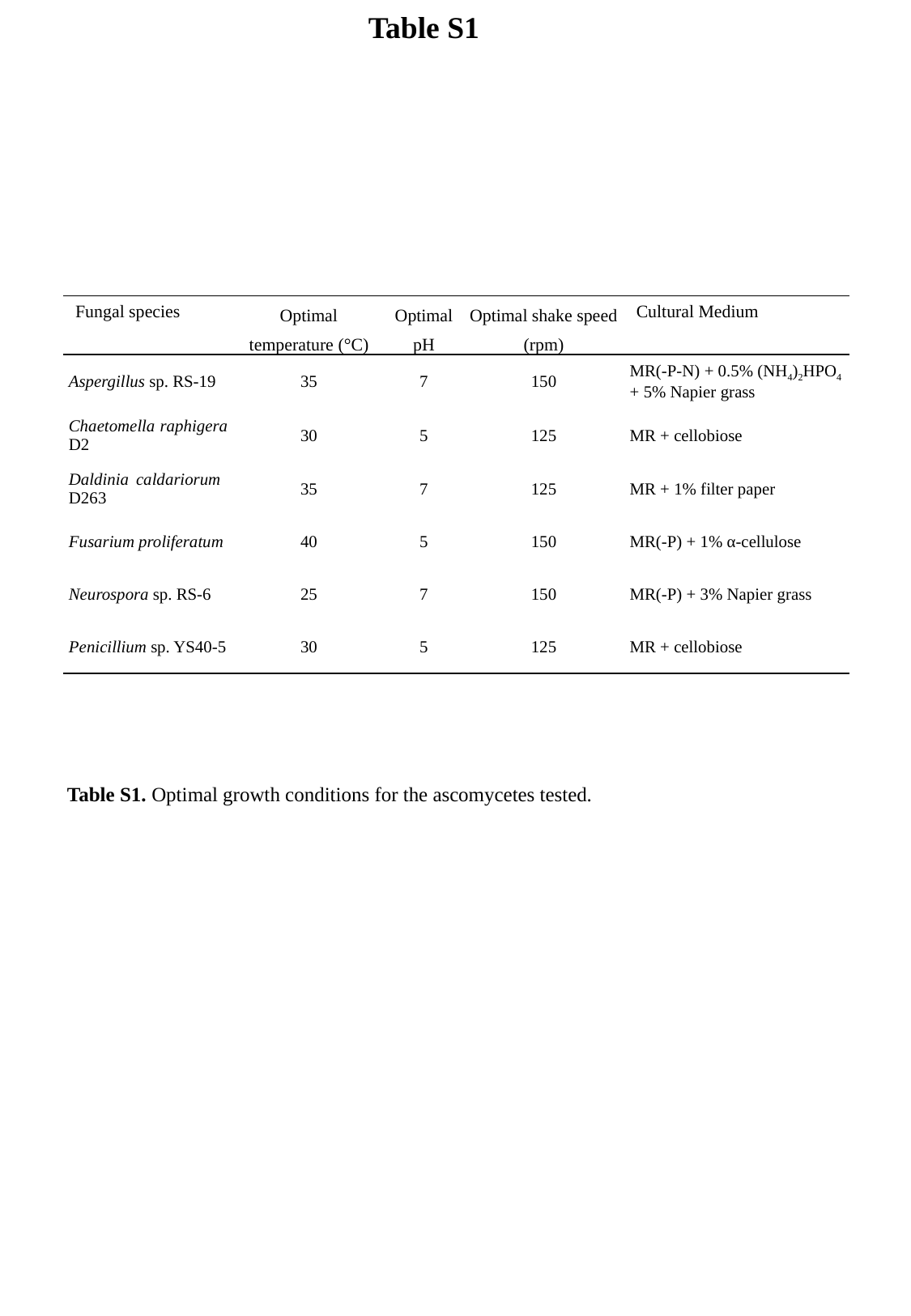

Table S1
| Fungal species | Optimal temperature (°C) | Optimal pH | Optimal shake speed (rpm) | Cultural Medium |
| --- | --- | --- | --- | --- |
| Aspergillus sp. RS-19 | 35 | 7 | 150 | MR(-P-N) + 0.5% (NH4)2HPO4 + 5% Napier grass |
| Chaetomella raphigera D2 | 30 | 5 | 125 | MR + cellobiose |
| Daldinia caldariorum D263 | 35 | 7 | 125 | MR + 1% filter paper |
| Fusarium proliferatum | 40 | 5 | 150 | MR(-P) + 1% α-cellulose |
| Neurospora sp. RS-6 | 25 | 7 | 150 | MR(-P) + 3% Napier grass |
| Penicillium sp. YS40-5 | 30 | 5 | 125 | MR + cellobiose |
Table S1. Optimal growth conditions for the ascomycetes tested.
