# Supplementary material for: From simple and specific zymographic detections to the annotation of a fungus Daldinia caldariorum D263 that encodes a wide range of highly bioactive cellulolytic enzymes
Source: Biotechnol Biofuels. 2021 May 21;14:120. doi: 10.1186/s13068-021-01959-1 (PMC8140500; doi:10.1186/s13068-021-01959-1)
Supplement: Supplementary file 2 — Additional file 2: Figure S1. D263 does not supplement the activity of CTec3. Figure S2. No D263 LPMO activity visualized on zymography. [file 13068_2021_1959_MOESM2_ESM.pptx]

## Slide 1
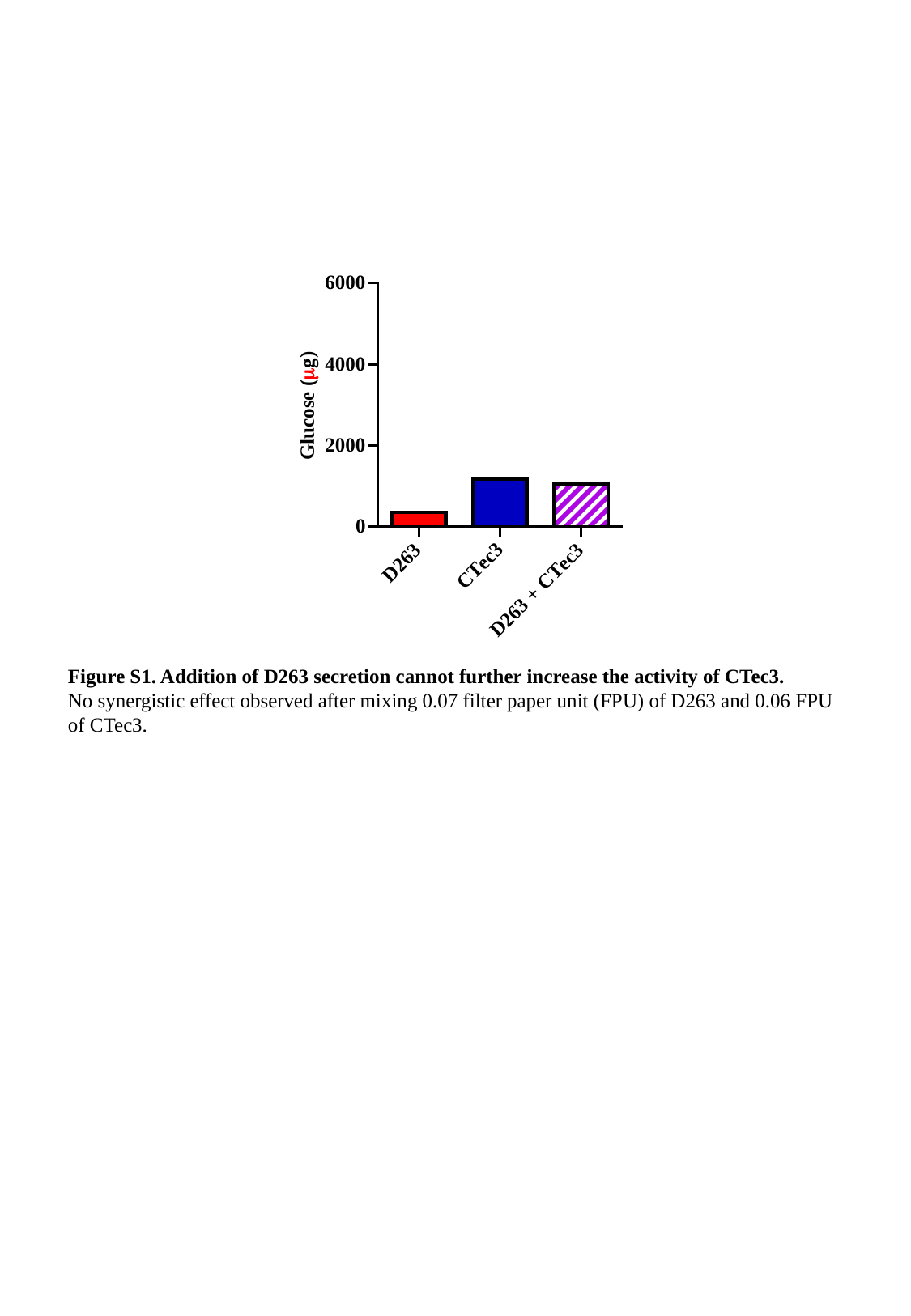

Glucose (mg)
Figure S1. Addition of D263 secretion cannot further increase the activity of CTec3.
No synergistic effect observed after mixing 0.07 filter paper unit (FPU) of D263 and 0.06 FPU of CTec3.

## Slide 2
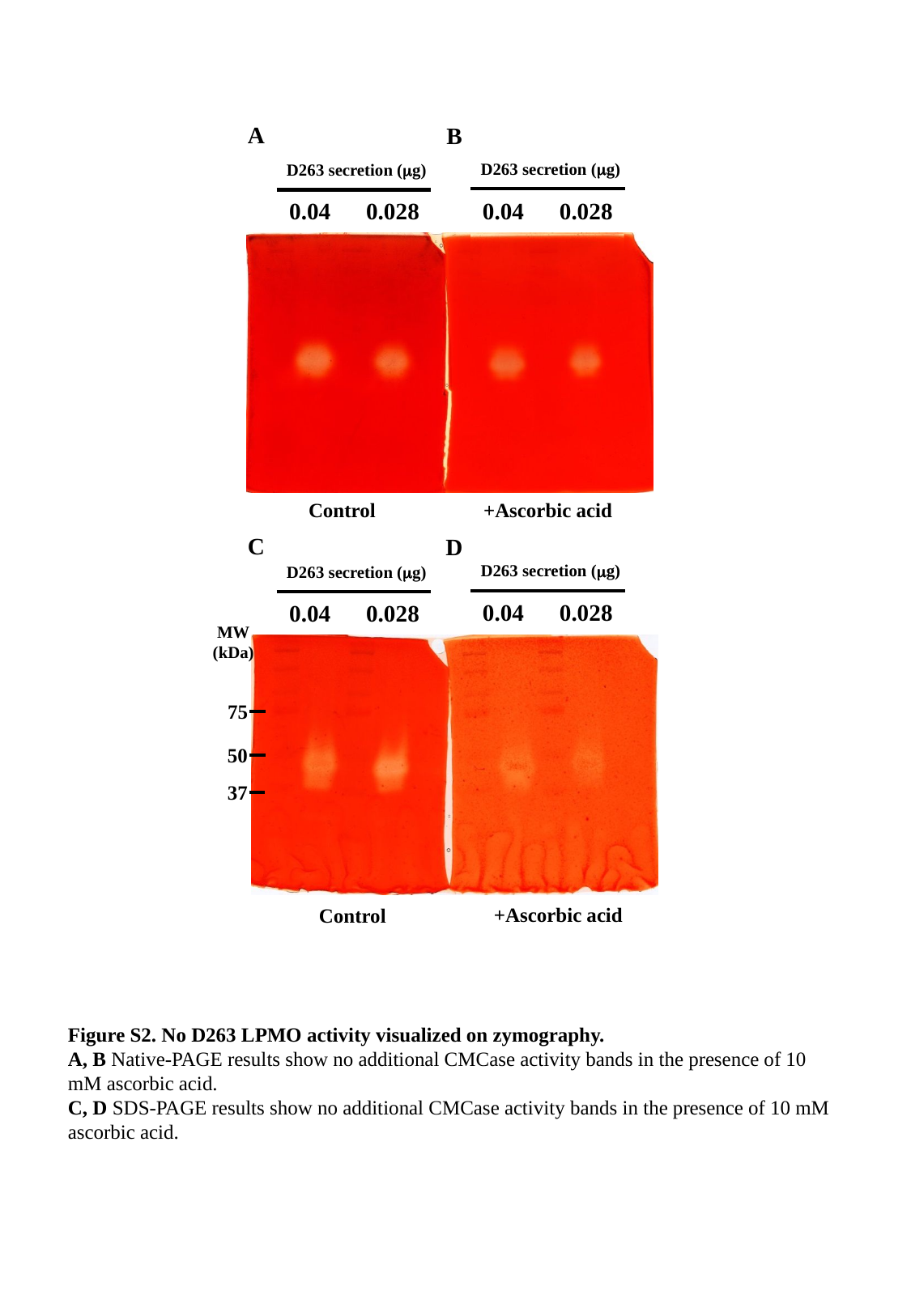

A
B
D263 secretion (mg)
D263 secretion (mg)
0.04 0.028
0.04 0.028
+Ascorbic acid
Control
C
D
D263 secretion (mg)
D263 secretion (mg)
0.04 0.028
0.04 0.028
MW
(kDa)
75
50
37
+Ascorbic acid
Control
Figure S2. No D263 LPMO activity visualized on zymography.
A, B Native-PAGE results show no additional CMCase activity bands in the presence of 10 mM ascorbic acid.
C, D SDS-PAGE results show no additional CMCase activity bands in the presence of 10 mM ascorbic acid.
